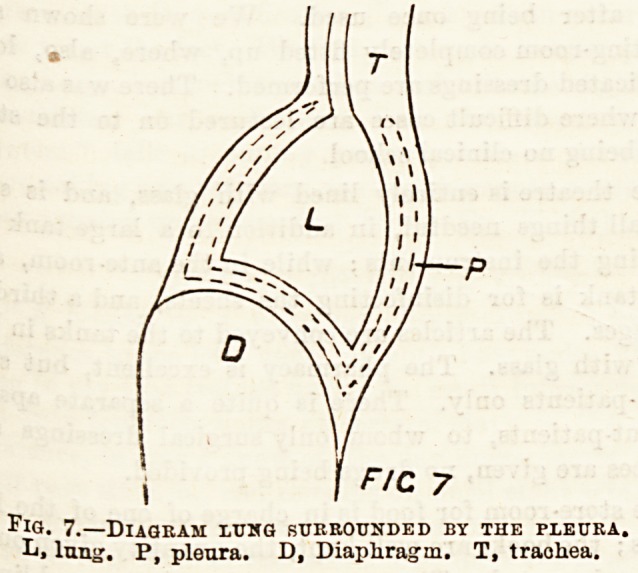# The Hospital Nursing Supplement

**Published:** 1895-09-28

**Authors:** 


### "The Hospital" Nursing Mirror.

Being the Extra Nursing Supplement of "The Hospital" Newspaper.

[Contributions for this Supplement should be addressed to the Editor, The Hospital, 428, Strand, London, W.C., and should have the word "Nursing" plainly written in left-hand top corner of the envelope.]

### News from the Nursing World.

### WHAT HAS BEEN DONE FOR NURSES?

When a man like the Hon. Sydney Holland, who, as is well known, devotes much personal attention to the welfare of hospital workers, asserts that "very little is done" for the comfort of nurses, it is natural to find the same idea in other quarters. Yet the picturesque theory that nurses' lives are shorter than those of other people is somewhat discredited by the number of nurses who apply for annuities, or who are in receipt of pensions. Even in respect of general health, it is probable that comparison between a hundred nurse-probationers and a hundred women of the same age and class, engaged in other callings, would be decidedly in favour of the former. They alone get free medical attendance and nursing when necessary, and the instruction they receive on the laws of health enables them to escape many minor ailments to which ignorance subjects other women. If nurses work too long hours, the blame rests on individual committees alone. The weekly half-day and monthly day and night out of hospital are certainly practical forms of extra "time-off." Nurses' average food is now good, plain, and wholesome, its variety depending on the skill of each housekeeper. At the Westminster all nurses have separate rooms, and at St. Thomas's the staff nurses' cubicles have been converted into separate apartments, and new rooms and sitting-rooms have been added quite recently, the Nightingale probationers being admirably lodged. At King's College Hospital and Charing Cross want of space still necessitates the use of cubicles, but these are well contrived, bright, airy little snuggeries. At St. Marylebone Infirmary nurses and servants have all separate bed-rooms, and St. Pancras Infirmary has an excellent new nurses' home. The fever hospitals lodge their nurses well, separate bed-rooms being provided. The Temperance and Mildmay Hospitals possess excellent nurses' rooms, and so does the New Hospital for Women, and new quarters will be ready at St. George's Hospital by and by; whilst many more institutions, the Poplar Hospital to wit, have done more than "a very little" for their nurses. The London Hospital has one excellent nurses' home, and for an additional wing a sum of £9,000 has been recently voted. The fact that the outlay in this direction has curtailed the dimensions of the Andrew Clark Memorial wing shows that nurses' comfort is not always a secondary consideration in hospitals. Mr. Holland, in his excellent letter to the *Daily News*, urges the appointment of younger men on committees, a step which should be of material benefit if those selected have administrative knowledge. Of most of the letters recently appearing on the great nursing question in the daily press, it is unnecessary to speak here, but they have doubtless served their turn as "copy" in the dull season of the journalistic year.

### THE PAYING PROBATIONER.

Although the number of probationers who pay for their training is very small in comparison with those who receive remuneration for their work, the former appear to monopolise an undue share of grievances. Either they are affronted because they have the same hours, rules, and duties as their paid sister nurses, or grieve because the privilege of shortened time on duty deprives them of experiences enjoyed by the others. In some extreme cases it is even asserted that the paying probationer is the drudge of the ward. Most of these real and imaginary grievances seem to have fallen to the lot of unsuccessful probationers. Perhaps those who succeed have less time for fault-finding. Considering the few paying probationers in the nursing world it is satisfactory to note that although the minority return home discomfited, a majority become excellent nurses. If their own private means or other circumstances justify them in paying for a full term of training, this fact has not been found detrimental to their earning a good certificate. It would therefore be an obvious injustice to many noble women to class for a moment all paying probationers with a few "three months' failures."

### POPLAR AND STEPNEY SICK ASYLUM.

The managers of the Poplar and Stepney Sick Asylum have issued a very full report of their district, combining with it an interesting account of recent progress in the nursing department. Abundant evidence is given of a sincere desire on the part of the authorities that their nurse-training school shall be on the best possible lines. In Miss Hannaford the managers have secured an able matron, and they appear desirous of forwarding her work of organisation. It has been decided that the title of "Sister" shall replace that of head nurse, and instead of "assistant nurses," the more convenient terms of "staff-nurse" and "probationer" are in use. The course of training, which covers three years, appears to be thorough, and the rules for the three grades of nurses are reasonable ones. In the time table, which forms part of the report, a weekly half-day off duty is allotted to each day nurse and a whole day once a month. The latter, however, is only reckoned from ten a.m. to ten p.m., which suggests that the ordinary early morning work precedes the holiday. No doubt those who are doing so much to promote the welfare of the nursing staff will alter this plan on reflecting how largely the pleasure of a "day off" is discounted to the nurse who has to do two or three hours' work before beginning it.

### ASYLUM ATTENDANTS AND NURSES.

The next examination of the Medico-Psychological Association is announced to take place on Monday,

November 4th. It is necessary for intending candidates to enter their names before Monday, October 7th, and they can obtain forms for the purpose by writing to Dr. Spence, Burntwood Asylum, Lichfield, which they should do with as little delay as possible.

### NURSING AT HALIFAX.

In the new infirmary at Halifax the nurses' quarters have been duly considered, and even in their present unfinished condition good promise of many separate bedrooms, of a library, and cheerful sitting-rooms is given. The position of the Nurses' Home is an ideal one, commanding beautiful views of the moors, and with such advantages of fresh air and sanitary surroundings as should make perpetual good health the leading characteristic of the nursing staff which will be installed there. The wards in the old buildings at present in use, are beautifully kept, and the condition of the laundry, linen-room, and kitchen speaks well for the administrative abilities of the matron. The children's ward is a little apart from the main building, and much care and skill fall to the lot of the little creatures who are tenderly nursed there. The most cheerful spot in the infirmary is, naturally enough, the boys' ward, and if the youngsters under treatment are fair samples of the juvenile population of Halifax, that town is to be congratulated on the good looks and pleasant manners of its citizens.

### AN UNDER-WORKED NURSE!

A correspondent writes from a pleasant town in England, complaining of having too much time on her hands. She is a private nurse, and says she has more hours off duty than she knows what to do with. The novel character of this grievance would be amusing if the nurse did not also speak slightingly of her patient as "old and uninteresting," and "likely to be a long case." Taking these remarks seriously, as we presume that our correspondent intends us to do, we advise her to devote a little time and thought to bringing some brightness into the dull life of her patient, whose age and illness should command sympathy, not contempt. Surely there is some branch of study which a nurse can take up? If she prefers needlework to reading or drawing, we should suggest her making useful garments for the sick poor. Materials at moderate prices are within most people's reach; and there are places, within a moderate walk, for her to visit. She might profitably make a tour of hospitals and other institutions in the town, thereby enlarging her interests, and gaining useful information. We advise our correspondent to think first of her patient, secondly of what she can do to help others, and only in the last place of herself and her grievances. We think if she has adopted nursing as a profession there will be many days in the future when she will look back with regret and longing to leisure hours which she professes to find it now difficult to fill.

### AMBULANCE INSTRUCTION FOR SEAMEN.

Two hundred and thirty merchant sailors and a number of other guests assembled at the Missions to Seamen Institute at Poplar, on the anniversary of its opening. The building was prettily decorated, an excellent concert was given, and the presentation of St. John Ambulance certificates took place. In the intervals between their voyages, many men have already received partial instruction in first aid to the injured from Dr. Radford, of the London Hospital, assisted by Mr. Noble. Fifty-nine of these sailors have completed the prescribed course of ambulance instruction, passing successfully the examinations held by Dr. Tunstall, of the volunteer forces. Already many occasions have offered for putting this useful training into practice, for severe accidents occur on board ship, such as dislocations, fractures, scalds in the boiler-room, bad cuts, &c. In the absence of medical aid, the seamen have frequently practised "second aid" also. On one occasion a certificated seaman is said to have instructed his shipmates how to apply splints to his own broken leg, with admirable results. The weekly classes are very popular amongst sailors, who realize the advantages derived from such teaching. It is suggested that a class for nursing should also be established.

### AN UNMANAGEABLE PATIENT.

In reporting the recent attempt at suicide of a patient at the Middlesex Hospital, the Press draws attention to the courage of the nurse who was in charge of him. She, with the assistance of another patient, did all that was possible to hinder the unfortunate man's determined attempts at self-destruction. His sudden rush for the window was in no way anticipated, and the presence of mind of the nurse delayed, though it did not prevent, the fall which resulted in serious injuries.

### VISITS TO IRISH HOSPITALS.

Many visits have been already paid to the Dublin hospitals by Her Excellency the Countess of Cadogan, who exhibits a very practical interest in the several departments of these institutions. The Convalescent home at Linden has also been inspected by the Viceregal party, and a promise made by Lady Cadogan to open the new wing of the Rotunda Hospital in November has given universal satisfaction.

### SHORT ITEMS.

The charge of discourtesy and harshness brought against a nurse in the out-patient department of the South Devon and East Cornwall Hospital has been authoritatively denied, but the father who made the accusation has again stated, through the medium of the local press, that his child was unkindly treated.—Party feeling runs high at Athlone on the question of nursing by nuns, with which we dealt last week. Many of the letters appearing in the Irish press acknowledge the necessity for nuns to undergo full nursing training before they are placed in charge of the sick in hospitals.—All the nurses who could be spared from the Newcastle Royal Infirmary on the 3rd inst. were entertained at luncheon by Sir George and Lady Trevelyan at Wallington Hall. A delightful day was spent in the beautiful grounds and in viewing the rare art treasures in the house. The nurses departed after tea laden with flowers and with photographs of this interesting Hall.—An annual subscription of twenty guineas has been voted by the Kensington Board of Guardians to the District Nursing Association in acknowledgment of valuable services to the sick poor in their own homes.

### Elementary Physiology for Nurses.

By C. F. MARSHALL, late Medical Registrar Hospital for Sick Children, Great Ormond Street.

### VIII.—THE RESPIRATORY SYSTEM.—(*Continued.*)

### THE MECHANISM OF RESPIRATION.

THE rhythmical action of respiration goes on unconsciously in ordinary quiet breathing about 15-18 times a minute. It is much quickened by muscular exertion, and is slower during sleep. It is much quicker in infants than in grown-up people. This rhythmical action differs from that of the heart in that the lungs themselves do not do the work but merely follow the chest wall. It is governed by a nervous centre, the respiratory centre, situated in the lowest part of the brain, the medulla. From this centre messages are sent to the various muscles concerned. The respiratory centre consists of two parts, one for inspiration, the other for expiration, and their activity is excited directly by the gases in the blood. Fixed respiratory activity, leading to excess of oxygen in the blood, causes cessation of respiration for a time, a condition known as apnœa.

We have assumed that the lungs are attached to the chest wall, but they are not really attached, for intervening between them is the pleura. This is a closed sac, one layer lining the chest wall, the other covering the surface of the lung. The two layers are for the most part in contact. The pleura may, in fact, be regarded as a closed bag, one side of which has been pushed in by the lungs.

The action of the pleura is as follows. The pressure of

FIG. 7.—DIAGRAM LUNG SURROUNDED BY THE PLEURA.
L, lung. P, pleura. D, Diaphragm. T, trachea.

air within the lung when at rest will be the atmospheric pressure, *i.e.*, 15 lbs. to the square inch. This pressure will tend to keep the lung against the chest wall, *i.e.*, to keep to the two layers of the pleura in contact. Counteracting this is the elasticity of the lungs themselves, the walls of which are constantly on the stretch, and tending to shrink away from the chest wall. This force is about ¼ lb. to the square inch, and is hence completely outbalanced by the pressure of the air in the lungs. Now, suppose a hole is made into the pleural cavity, air will enter and press directly on the surface of the lung. We have now a pressure of 15 lbs. tending to keep the lung against the chest wall, and a pressure of 15 lbs. and ¼ lb. tending to cause it to collapse, *i.e.*, the pressure on the outer surface of the lung is greater than on the inner, and hence the lung collapses. This is the effect of a knife wound of the chest wall penetrating the pleura, the lung collapses, and does not follow the movements of the chest wall as before.

In pleurisy or inflammation of the pleura the surfaces of the pleura are roughened, and friction occurs between them. This rubbing of the two surfaces may be heard with a stethoscope. In further stages of pleurisy serous watery effusion may accumulate to a great extent between the two surfaces, and press on the lung, causing partial collapse and hence laboured breathing. In pneumonia or inflammation of the lung itself, there is solidification of the lung tissue, and so difficulty of breathing, accompanied by high fever. Bronchitis is inflammation of the air passages from exposure to cold and irritant matters, and generally affects the larger bronchial tubes. Phthisis, or consumption, is associated with actual destruction of the lung tissue and the formation of cavities.

### CHANGES CAUSED BY RESPIRATION.

*Changes in the Air.*—Expired air differs from inspired in three chief respects. It is heated to the temperature of the blood; it is saturated with moisture; and it has undergone changes in composition. The quantity of moisture expired in a day is roughly about a pint, but may be more.

The gases of the air change thus:—

| | Oxygen. | Nitrogen. | Carbonic acid. |
|---|---|---|---|
| Inspired air... | 21 | 79 | 0·03 |
| Expired air... | 16 | 79 | 5·00 |

Thus the nitrogen is unchanged; there is a loss of about 5 per cent. of oxygen and a gain of 5 per cent. in the form of carbonic acid. About 18 cubic feet of oxygen are absorbed in the lungs per day, and about 18 cubic feet of carbonic acid given out. The carbonic acid is best estimated as charcoal, and amounts to eight ounces a day. Thus a man is a kind of human chimney constantly polluting the atmosphere, about 350,000 tons of carbon as carbonic acid being expired by the human population of the globe in a day, to say nothing of other animals as well.

If a man is put in a closed chamber the oxygen will be steadily diminished and the carbonic acid increased. This will after a time cause difficulty in breathing and ultimately death by suffocation. This fact was well illustrated in the memorable Black Hole of Calcutta. In such a case the blood is only imperfectly purified, and both arteries and veins are full of venous blood. This at first causes increased respiratory activity; then convulsions from the brain becoming affected; finally, unconsciousness and death.

Deficiency of oxygen is more important than excess of carbonic acid; for if air is replaced by nitrogen gas no accumulation of carbonic acid occurs, but in the absence of oxygen an animal is soon suffocated. On the other hand 10 or even 20 per cent. of carbonic acid may be present in the air without producing serious effect, if the quantity of oxygen is increased sufficiently. Hence the evil effects of overcrowding are obvious. The proper amount of space that should be allowed every man is 800 cubic feet, and this should be accessible directly or indirectly to the atmosphere.

### "The Hospital" Convalescent Fund.

### THE NURSE'S BED.

DELICATE or tired nurses to whom a visit to the seaside or country would be of permanent benefit, and who cannot afford to take a holiday at their own expense, are helped by this fund. On the recommendation of matron or doctor to whom their circumstances are known, arrangements are made to give the kind of change most likely to do them good. Applications should be addressed to the Hon. Secretaries of THE HOSPITAL Convalescent Fund, 428, Strand, London, by whom subscriptions are received and acknowledged.

### Where to Go.

THE NATIONAL UNION OF WOMEN WORKERS.—The seventh annual conference of women workers will take place at the Mechanics' Hall, Nottingham, on Tuesday, Wednesday, Thursday, and Friday, October 22nd, 23rd, 24th, and 25th when many interesting papers will be read and discussed.

### New York Training School for Nurses, Blackwell's Island.

ADDRESS TO THE GRADUATING CLASS.

In his recent address to the graduating class, Dr. Frederick Holme Wiggin, Visiting Surgeon to the City Hospital, gave much good advice to the nurses of the training school, a great deal of which might well be taken to heart by their sisters in the profession all over the world, more especially by those who may adopt private nursing as their particular line. Speaking from a large experience, Dr. Wiggin declared his conviction that much of the success which has crowned medical efforts of late years has been due to the intelligent assistance of the trained nurse, and expressed it as his opinion that the graduates of the New York City Training School were the equals of any in the world. "The first thing," said Dr. Wiggin, "which it is necessary for a nurse to do on taking charge of a case is to win the confidence of her patient, and the persons with whom she is brought in contact. . . . The nurse should impress on her patient's mind, by her actions rather than her words, the fact that it gives her pleasure to do anything that will add to his or her comfort. We all know of patients whose convalescence has been retarded by reason of the question constantly arising in their minds, Will or will not the nurse take offence if asked to do this or that service? . . . Remember that your position, no more than the physician's, can be affected for the worse by anything, however menial, it may be necessary at times for you to do to add to your patients' comfort, or to increase their happiness, and therefore their chance of a speedy recovery or convalescence. Be charitable . . ." Dr. Wiggin exhorted nurses to pay strict attention to their own health, and concluded with the following words:—Finally, do not make the fatal mistake of believing that your happiness is to be found outside and apart from your life's work, and that your chief reward is to be the material compensation which you will receive for your labour. If, unfortunately, this is the view you are taking with you as you enter upon this life of hard work (one possibly in many ways surpassing even the physician's in physical hardship and self-abnegation), there is little risk in predicting that yours is to be a life of hopeless drudgery, of bitter repining, and at last failure. If, on the other hand, you enter on this life with a higher ideal, a desire not only to earn an honourable support, but to follow somewhat in the footsteps of the Master, with a determination always to keep in mind the golden rule of "doing unto others as you would they should do unto you," to subordinate your personal comfort to that of others, to find your greatest happiness in doing whatever comes to hand faithfully and well, then will you find as time passes that your most valued reward will be the smile of gratitude and pleasure the mother greets you with as you return to her the babe so recently at death's door, and now largely by your intelligent watchfulness restored again to health, or in the prayer of thanksgiving in which blessings are asked for you, offered by the wife, it may be, for the return of health to the husband and father, who, having met with some accident, has been obliged to undergo a serious operation, which the surgeon might have found difficult to perform successfully without your valuable aid. Then, I repeat, you will find your greatest reward in the proud consciousness of duty well performed. Be true to your Alma Mater, to your patient; to your physician for the time being, and to yourselves, and the result cannot be long in doubt. Success must be yours; and your lives cannot fail to be a daily benediction to the communities, be they large or small, in which you live and move and have your being.

### Nursing in Rome.

II.—THE CONSOLAZIONE HOSPITAL BY THE FORUM.

It was entirely through the kindness and influence of Lady Dufferin that we were allowed to visit this and other hospitals in Rome. Dr. Prochet fixed the hour of seven in the morning for our inspection, as the doctors then made their first round, and we should see the work in full swing.

The hospital is maintained by ancient legacies for the reception of every kind of accident; it contains eighty beds for men, thirty for women, and a large children's ward. It is served by seventeen Sisters, three of whom are Maltese, under six doctors and there are a few students.

All the wards were bright and cheerful, though the occupants were suffering from terrible accidents, some, however, being examples of almost miraculous cures. The children's ward was specially bright with palms and flowers, the first we had seen in an Italian hospital.

Each ward was supplied with a covered handcart for conveying the patients about the building, also with thick glass tables and large baskets of well-rolled bandages.

The tables at the sides of the children's cots had two tiers made of glass. Many of the patients were very handsome, and their faces curiously pathetic.

Belonging to the hospital is an isolated ward for erysipelas, a mortuary, and a laundry. Dressings are burnt in a special stove after being once used. We were shown a small operating-room completely fitted up, where, also, long and complicated dressings are performed. There was also a small room where difficult cases are lectured on to the students, there being no clinical school.

The theatre is entirely lined with glass, and is supplied with all things needful, in addition to a large tank for disinfecting the instruments; while in the ante-room, another huge tank is for disinfecting the sheets, and a third is for bandages. The articles are conveyed to the tanks in baskets lined with glass. The pharmacy is excellent, but supplies the in-patients only. There is quite a separate apartment for out-patients, to whom only surgical dressings and appliances are given, no drugs being provided.

The store-room for food is in charge of one of the Maltese sisters; the books are well kept, the quantity given out being accurately noted. The store-room for clothes and linen was in perfect order, and well stocked with articles made by the Sisters, and bearing abundant testimony to their industry.

Friends of the patients are allowed to come twice a week, but are strictly forbidden to bring in any eatables except fruit. We were very pleased with this hospital, seen under the guidance of Dr. Prochet; it is modern in its arrangements, and well supplied with everything needful.

### Royal British Nurses' Association.

The Secretary of the Royal British Nurses' Association is most anxious that the attention of all members should be drawn to the notice that the lending and reference library will be open on and after October 1st for the use of members, at the offices, 17, Old Cavendish Street. Books can be obtained and exchanged daily from ten to four, Saturdays ten to one. The rules sanctioned by the committee were published *in extenso* in the August number of the *Nurses' Journal*, and a copy will be found pasted in each volume issued from the library.

### Everybody's Opinion.

[Correspondence on all subjects is invited, but we cannot in any way be responsible for the opinions expressed by our correspondents. No communications can be entertained if the name and address of the correspondent is not given, or unless one side of the paper only be written on.]

### "THE OCKLEY SYSTEM."

MISS BERTHA BROADWOOD writes: An article under the above title in a recent issue of your paper has been brought to my notice. As originator of the Ockley system of cottage nursing, now widely spread beyond my own neighbourhood, and as promoter of training fields specially adapted for cottage nurses, I beg you kindly to read and then forward to the writer of the article the marked portions of the accompanying copy of the third edition of "Nurses for Sick Country Folk," sold at the office of the Affiliated Benefit Nursing Associations, 12, Buckingham Palace Road. My pamphlet contains information up to a year ago regarding cottage nurses, and a more accurate description of the training they now receive, and of the training which I aim at for them, than the article in your paper and the letter it referred to gave. Evidently your contributor has no personal knowledge of cottage nurses or their qualifications, and it would give me great pleasure if he or she would come and spend a few days here to make the acquaintance of some of the Ockley nurses, and learn the estimate in which they are held by the doctors under whom they work. May I ask you to tender this invitation with the pamphlet, and also to give publicity to this letter in your columns?

[*** We fail to see on what evidence Miss Broadwood assumes that we have "no personal knowledge of cottage nurses or their qualifications," and we can assure her that such is not the fact. We find nothing in the pamphlet which she was good enough to enclose which in the slightest degree invalidates our contention that the so-called "Ockley System" fails to supply properly-trained district nurses, even taking as a standard the requirements exacted for outdoor nursing under the Poor Law.—ED. *T. H.*]

### NURSES' HOURS ON DUTY.

"A NURSE IN A COUNTRY INFIRMARY" writes: With reference to the long hours of work now under discussion, I beg to say that trained nurses in small infirmaries are often on duty fourteen hours on several days in the week. For instance, in one I know of, the day nurse comes on duty at half-past six a.m., and remains on until eight p.m.; the only time she is out of her ward being for her meals. This is the case on four days of the week. Two days she is off duty from half-past five p.m. until half-past nine p.m., and the same every alternate Sunday. The night nurse has to relieve the day nurse on all these occasions. Nursing in small infirmaries is, I think, more fatiguing and trying than in any hospitals, for there are so many old and helpless patients.

### FIRST AID.

A LADY writes: On September 13th, whilst on a visit to Conway Castle, North Wales, I had the opportunity of witnessing a most plucky and praiseworthy act on the part of a hospital nurse in rendering "first aid to the injured." An accident happened on the Conway Suspension Bridge. A cart, crossing sharply round a curve, overturned, and without warning an old man and a lad were thrown violently to the ground. The lad, who lost all consciousness, was quickly removed to the doctor's house; no one seemed to know what to do for the old man, who was in an agony of pain, when a young lady came quickly upon the sad scene. She seemed to take in the whole situation immediately. Removing a silk handkerchief from the old man's neck, she made a "tourniquet" for his leg, which arrested the flow of blood which was issuing forth from the broken part of the limb. With sticks and handkerchiefs she formed an impromptu splint which kept the leg as straight as possible. The sufferer was then taken to the doctor's house at Conway, and he, with the aid of this willing nurse, applied an additional splint, and the patient was removed to the Llandudno Cottage Hospital, where he is going on well, and the doctor hopes to save the leg. The young lady received her training at the Bristol Royal Infirmary, and I heartily congratulate that institution upon having taught her so efficiently. There is no doubt that she was the means of saving the old man's life.

### NURSES, THEIR UNIFORM, AND THE PUBLIC.

A NURSE writes: Your article in this week's HOSPITAL, "Nurses, their Uniform, and the Public," reminds me of a very uncomfortable moment which occurred to me at the Langham Hotel not long ago. I was asked to breakfast there by an uncle who had come up to London, and was staying there. On my arrival (in uniform, of course), much to my surprise and the indignation of my uncle, the manager informed me that no nurse in uniform was allowed to have any meals in the hotel. After considerable protestation on the part of my uncle, the manager allowed me to remain only on condition that I removed my cloak, bonnet, and apron. Where a nurse is compelled always to wear uniform during hospital life it is hard that she may never be able to see friends and relations who may happen to be staying in hotels.

[Our correspondent's experience enforces the views which we expressed last week. Nurses should only wear uniform when on duty, or engaged in their ordinary avocations. In private life, or when on pleasure bent, uniform tends to make the individual conspicuous, and therefore uncomfortable, as it is not suited to all the circumstances of private life.—ED. *T. H.*]

### NURSING IN A FEVER HOSPITAL.

"A LATE CHARGE NURSE" writes: Having spent two years at a fever hospital under the Metropolitan Asylums Board, and knowing the arrangements on the small-pox ships to be much the same, I hope to be allowed to say something about the nurses. The charge nurse was responsible for two wards, one containing twenty beds of acute cases, the other thirty convalescents (the latter often with "complications"). In each ward there was a "first assistant," or trained nurse, who shared with the charge nurse the responsibilities of the actual nursing. The second assistant, *i.e.*, probationer, saw all that was done, and was able in many little ways to save the time of the two senior nurses. No nursing was entrusted to her, but she proved at times a most invaluable helper. Nursing was nursing pure and simple at that hospital, because the charge and first assistant had only nursing to do. With the many complications of fever—the surgical cases sent from general hospitals, mothers with babies perhaps only a day old—there was more than enough to do. It was splendid work altogether. Of course the isolation was great, and there was none of the excitement of visitors, and no one to admire the "devoted" nurses, yet we were happy, and could not help being so with an excellent matron and medical staff, who did all they could for our comfort. I would back that place against any hospital in England for good up-to-date nursing. A probationer enters a general hospital with no previous training, and at the end of a month finds herself entrusted to take part in the nursing as well as sweeping and dusting the ward, and from the day of her arrival she is called "nurse"!

### TEA STAINS.

"AN OLD NURSE" writes: I shall be very thankful to any one for telling me how to take the stain of tea from a blanket. A teapot was completely upset, and although the spot was washed directly and the blanket washed twice since the stain remains. I shall be grateful for advice through THE HOSPITAL, which I have taken in for many years.

### The Book World for Women and Nurses.

[We invite Correspondence, Criticism, Enquiries, and Notes on Books likely to interest Women and Nurses. Address, Editor, The Hospital (Nurses' Book World), 428, Strand, W.C.]

Thrown Away; or, Basil Ray's Mistake. By Nat Gould. (London: George Routledge and Sons. 1894.)

This tale is extremely good so long as it only deals with thrilling incident, sport, and adventure, but as a study of human nature, especially of women, it is a complete failure. The two prominent female characters of the book, namely, the half sisters, Lilian Preston and Ada Gosper, are far fetched to a degree. We have but little sympathy with either. Lilian is unnaturally wicked and Ada preternaturally good; both are essentially unreal. Basil Ray ought to have been a hero of the Guy Livingstone order, but as he does not attain to such a height he is rather in a wrong position. Lilian, however, is in love with him, and this same love leads her into direful extremities, she manages to shoot her husband on pretext of mistaking him for a burglar; though that is only her refuge at the last, as for some time the crime remains undiscovered, and she marries Basil Ray. The burglar who saw her do the deed is brought to bay, and then ensues a truly dramatic scene in a court of law, though at the end of it the readers cannot quite ascertain whether she did murder Preston intentionally or not; but that seems of little consequence as she goes mad and dies, so cheating the hangman. Then comes on the good lady of the play who, after many impossible events, eventually becomes the second Mrs. Ray, and they live happy ever after, &c., &c. The racing jargon of the book is as excellent as the sentiment is sickly, and the graphic description of the winning of the Canfield Cup would stir the blood of any true lover of horse-flesh.

### A NEW LADIES' NEWSPAPER.

"Madame."

The question, Is there room for another journal? is a very old one, but since experienced publishers continue to introduce fresh competitors into the field we presume they know that the public are either as yet unsatisfied or demand continuous novelty. *Madame*, the new venture just launched in the newspaper world by the proprietors of *St. Paul's*, has determined to distance all rivals in its own particular field. The cover is most attractive in design, the printing and paper are excellent, and the whole journal is a most imposing production for the small outlay of sixpence. *Madame* evidently intends to assist her lady readers in all the most varied of their wants. An experienced expert has charge of each section, so that its advice may be sound and up-to-date. Illustrations are numerous, and nothing seems to have been overlooked which will prove of interest to the fair public *Madame* desires to please.

### MAGAZINES OF THE MONTH.

The Humanitarian for September is, if anything, an improvement upon the past numbers. There are many articles of both a topical and an interesting nature. The opening paper is on "The Social Condition of the Agricultural Labourer," by the Earl of Winchilsea. Lord Winchilsea's interest in all questions of an agricultural nature is too well known to require any commentary here; his efforts both in and out of Parliament to promote the interest of this particular class, and to benefit all persons connected with the land, are well recognized. Upon this subject he is able to talk from a full mind, and what he says is worth some study at our hands. A new departure in the work of the National Agricultural Union, founded by Lord Winchilsea, is a scheme for encouraging the employment of women by opening up for them suitable industries in rural villages when the winter season sets in. Further on in these pages is Mrs. Haweis' "The Golden Age," thoughtfully and charmingly written.

### Our American Letter.

It is probable that in time outdoor, or district nursing, will assume as great proportions here as it has already done in England. We have been dilatory in admitting its importance, or perhaps our nursing system has been altogether too young to include so large and important a departure. Most of the graduates have been required for the formation of new nursing schools. Private nursing has also demanded more recruits, as our physicians have learnt to depend in such a marked degree upon the aid of qualified nurses. But each year the increased number of training schools results in a better supply of trained nurses, hence due provision for the sick poor in their homes will soon be made in our larger American cities. Already some of the most accomplished superintendents of training schools are bestirring themselves in the matter, and their influence and teaching will secure a proper curriculum for those preparing to take up this useful branch of nursing.

As regards actual nursing news there is not much to record on account of the holiday season. The Superintendent of the Pittsburg Homœopathic Hospital, Miss Preston Wright, has resigned the post filled by her with distinction for over six years, on account of her approaching marriage. Her loss will be much felt in the training school, which owes so much of its success to her personal enterprise.

Dr. Spencer Kinney handed their diplomas to the graduates of the Middletown State Hospital in the presence of a number of the friends and relatives of the nurses. The badges were this year kindly provided by Dr. Talcott, the superintendent of the hospital.

Two months' probation precedes two years' training at the Virginia Hospital, and candidates under nineteen are not eligible for admission. The hospital contains fifty beds, and additional training is given in district as well as private nursing.

### Appointments.

[It is requested that successful candidates will send a copy of their applications and testimonials, with date of election, to The Editor, The Lodge, Porchester Square, W.]

The Borough Sanatorium, St. Helens.—Miss Edith L. MacCarthy has been appointed matron of this sanatorium. She was trained at the General Infirmary, Leeds, did two years' private nursing, and afterwards held the post of sister-in-charge of the scarlet fever block of the Hull Sanatorium for three years. Miss MacCarthy holds excellent testimonials, and we join our good wishes for her future success to those of many old friends, by whom her departure from Hull is regretted.

### Notes and Queries.

### Queries.

(251) *Outdoor or Temporary Nurse.*—Will you please tell me of any institution which would employ a married nurse without children? I have had a year's training in a general hospital and three years' experience in a large workhouse infirmary, besides private cases.—*Nurse C.*

(252) *Books.*—Kindly recommend a good book for a mother to take abroad as a guide to the care of her children.—*S. Gamp.*

(253) *Training.*—Can anyone advise me of the best hospital to go to for three months to work up knowledge which has grown rusty in the country?—*Stagnation.*

(254) *Climate.*—Will you advise me of bracing places in Devon or Somerset?—*Nurse.*

### Answers.

(251) *Outdoor or Temporary Nurse* (*Nurse C.*).—We do not think you will have any difficulty in getting work, if your testimonials are good ones. Answer advertisements.

(252) *Books* (*S. Gamp*).—"Help in Sickness and to Health," published by the Scientific Press.

(253) *Training* (*Stagnation*).—Few London hospitals would take you for three months; but you had better write to the matrons of those who do, and to some of the good provincial hospitals. You will find a list in "Burdett's Hospital Annual."

(254) *Climate* (*Nurse*).—There are charming places on the north coast of both counties which would suit you. Do not select a small bay, but there are open spots in plenty to choose from.

## Figures and Tables

**Fig. 7 f1:**